# Distance Calibration between Reference Plane and Screen in Direct Phase Measuring Deflectometry

**DOI:** 10.3390/s18010144

**Published:** 2018-01-06

**Authors:** Shujun Huang, Yue Liu, Nan Gao, Zonghua Zhang, Feng Gao, Xiangqian Jiang

**Affiliations:** 1 School of Mechanical Engineering, Hebei University of Technology, Tianjin 300130, China; hbhsj824@163.com (S.H.); Yue.Liu@hud.ac.uk (Y.L.); ngao@hebut.edu.cn (N.G.); 2 Centre for Precision Technologies, University of Huddersfield, Huddersfield HD1 3DH, UK; f.gao@hud.ac.uk (F.G.); x.jiang@hud.ac.uk (X.J.)

**Keywords:** distance calibration, direct phase measuring deflectometry (DPMD), specular surface measurement, phase measurement, three-dimensional (3D) sensing

## Abstract

The recently developed direct phase measuring deflectometry (DPMD) method can directly measure the three-dimensional (3D) shape of specular objects with discontinuous surfaces, but requires a calibrated distance between a reference plane and liquid crystal display screen. Because the plane and screen are different distances from the imaging device, they cannot be clearly captured given the limited depth of field (DOF) of the lens. Therefore, existing machine vision-based methods cannot be used to effectively calibrate a DPMD system. In this paper, a new distance calibration method that uses a mirror with a hollow ring matrix pattern and a mobile stage is presented. The direction of the mobile stage in the camera coordinate system is determined by the mirror’s pattern at several positions in the camera’s DOF so that the reference position outside of the DOF can be calculated. The screen’s position can also be calibrated by displaying patterns at a known scale. Therefore, the required distance is accurately obtained in the camera coordinate system. Evaluation results show that the maximum value of the absolute error is less than 0.031 mm. The experimental results on an artificial stepped mirror and a reflected diamond distribution surface demonstrate the accuracy and practicality of the proposed method.

## 1. Introduction

Optical three-dimensional (3D) shape measurement techniques have been widely applied in many fields such as reverse engineering, biological recognition, and the digitalization of cultural relics. Among them, structured light pattern projection is a popular method because of its advantages of full-field acquisition, fast and automatic data processing, noncontact operation, and high accuracy [1,2,3,4,5]. However, most of the existing techniques can only be used to measure objects with a diffuse surface. With the advent of intelligent manufacturing, there are many specular objects in actual industrial applications. The research of shape measurement for these objects is still at an early stage. The main methods of measuring the 3D shape of specular objects use a coordinate measuring machine or change the surface characteristics using spray paint, which destroys the surface properties and is unacceptable in many fields. Therefore, it is important to study optical 3D full-field shape measurement methods for objects with a specular surface.

Phase measuring deflectometry (PMD), or the fringe reflection technique, has been widely studied for measuring specular free-form surfaces because of its advantages of noncontact operation, full-field measurement, fast acquisition, high precision, and automatic data processing [6,7]. PMD has been applied to measure aspheric mirrors and dynamic specular surfaces, detect subsurface cracks, and has been used on specular surfaces from micro-scale to large scales [8,9,10,11,12,13]. For example, aspherical and spherical mirrors, silicon wafers and ball grid array, automobile industry (automotive glass, car body) [14]. This technique must display straight sinusoidal fringe patterns on a liquid crystal display (LCD) screen or project a structured pattern onto ground glass. From a different viewpoint, the fringe patterns reflected on the measured surface appear deformed with respect to the slope variation of the specular surface. The modulated fringe patterns can then be captured by an imaging device such as a charge-coupled device (CCD) camera. The phase information in the deformed fringe patterns is then demodulated to obtain the slope of the measured specular surface and the 3D shape of the tested surface can then be reconstructed by integrating the gradients [15,16,17]. 

Lee et al. used the Ronchi test to measure the aspheric surfaces [18]. The Ronchi test provided information about the surface but the ronchigrams represented slope errors. Su et al. applied the developed PMD method called software configurable optical test system (SCOTS) to measure an X-ray mirror with a precision and accuracy better than 100 nrad (RMS) and ~200 nrad (RMS), respectively [19]. Fang et al. have reviewed current research on manufacturing and measuring freeform optics [20]. Häusler et al. introduced a novel microdeflectometry technique to measure the microtopography of specular surfaces by using a micro-objective [9]. The lateral resolution was better than 1 µm and the height resolution was in the range of 1 nm. Krey et al. presented a fast optical scanning deflectometer to measure the topography of large silicon wafers [21]. Skydan et al. applied the non-full-field reflective technique to measure automotive side glass to speed up and ensure product development and manufacturing quality [22]. Höfling et al. presented a new method for measuring reflective objects by using phase information and applied it to detect shape defects on car body sheets [23]. The surface curvature and then the discrimination of defects were directly calculated from the phase data. Quantitative height information could also be derived from the surface curvature by using two integration steps. Owing to the integration procedure, complicated specular components with isolated and/or discontinuous surfaces cannot be obtained from the measured phase data. The above PMD methods therefore only measure the local slope instead of the actual 3D shape of smooth surfaces.

To solve the above problem of measuring complicated specular surfaces, the direct PMD (DPMD) method has recently been developed to obtain the full-field 3D shape of specular objects with isolated and/or discontinuous surfaces [24,25]. This method directly determines the relationship between the absolute phase and depth information. To measure accurate 3D shape data, it is an important step for PMD to calibrate the system parameters, including the distance *d* between the reference plane and an LCD screen and the distance between the reference plane and a CCD camera [26,27]. Among them, *d* is an important parameter for the 3D shape measurement of specular objects. However, the existing calibration methods cannot calibrate *d* accurately. In DPMD, a mirror with a hollow ring matrix pattern is used as the reference plane position. The mirror and LCD screen cannot be clearly imaged by the CCD camera simultaneously because they are different distances to the CCD camera and the imaging lens has a limited depth of field (DOF), which means that one of them will be out of the DOF of the imaging lens. Some researchers used an infinite focus measurement machine (IFM) to obtain dataset at a high depth of focus [28,29]. IFM is based on the focus-variation technique, which combines the traditional surface metrology and the microcoordinate measurement technology. Therefore, existing methods cannot calibrate *d* accurately [25].

This paper presents a novel distance calibration method to determine parameter *d* between the reference plane and LCD screen with the aid of an accurate moving stage. In this method, the mirror with the hollow ring matrix pattern is placed on the stage and can be accurately translated to different positions. Within the camera lens DOF, the mirror is moved to several known positions and the hollow ring matrix pattern is clearly captured by the CCD camera at each position. The center of each hollow ring marker on the mirror can be extracted from these captured images. The moving direction of the stage in the camera coordinate system is determined using the extracted markers and internal parameters of the CCD camera. Therefore, the orientation of the mirror can be accurately determined in the camera coordinate system, even when it is out of the DOF of the CCD camera. Meanwhile, the position of the LCD screen can be calibrated in the camera coordinate system by generating known-size patterns using software, for example, checkerboard or hollow ring matrix patterns, on the LCD screen. Therefore, the distance *d* between the mirror at the reference position and the LCD screen can be accurately determined in the same camera coordinate system. The experimental results of direct and indirect measurements show that the proposed calibration method can accurately obtain the distance between the reference position and LCD screen. The proposed calibration method yields an accurate distance so that the DPMD system can accurately measure the 3D shape of specular objects with isolated and/or discontinuous surfaces. 

In the rest of this paper, Section 2 describes the principle of the proposed distance calibration method, including a brief introduction to the DPMD system, calculation of the direction of the moving stage, and distance calculation. Section 3 shows some experimental results with respect to error analysis and the actual 3D measurement of specular objects with multiple isolated and/or discontinuous surfaces. The conclusions and remarks regarding future work are given in Section 4.

## 2. Principle

**Figure 1:**
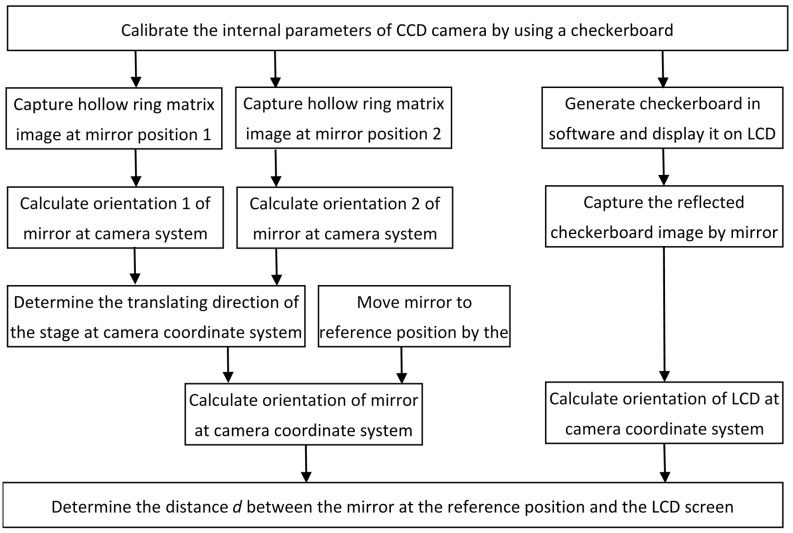
Flowchart of distance calibration using a moving stage in the direct phase measuring deflectometry (DPMD) system.

The proposed distance calibration method consists of several steps, as shown in Figure 1. Section 2.2 describes the details of each step. The direct relationship between absolute phase and depth is first briefly described in Section 2.1, so that this paper is self-contained. 

### 2.1. Direct Relationship between Absolute Phase and Depth

**Figure 2:**
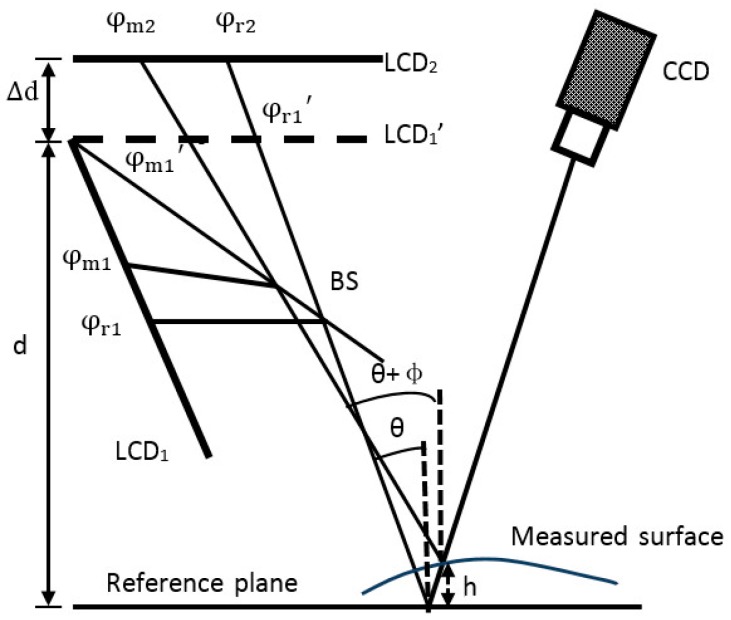
Schematic of the 3D measurement system based on DPMD.

A schematic diagram of the process to measure specular objects with isolated and/or discontinuous surfaces is shown in Figure 2. There are two LCD screens called LCD_1_ and LCD_2_, a CCD camera, and a plate beam splitter (BS). The BS implements a parallel design with the two LCD screens and thus avoids the need to mechanically move a screen to two positions during the calibration and measurement procedures. As illustrated in Figure 2, two rays of light are displayed and reflected into the CCD camera via the measured surface and mirror at the reference position. The two incident rays correspond to the same reflected light. The absolute phases of the two incident rays are denoted $\varphi_{r1}$ (or $\varphi_{r1}\prime$) and $\varphi_{r2}$ on the reference plane and $\varphi_{m1}$ (or $\varphi_{m1}\prime$) and $\varphi_{m2}$ on the measured specular surface. Angles *θ* and *θ* + *Φ* are the angles between the incident ray and normal vectors of the reference and measured specular surfaces, respectively. The period of the displayed fringe pattern on the LCD screen is denoted by q. Moreover, ∆*l* is the distance on LCD_1_′ between the two incident rays because of the height and gradient of the measured surface. Parameter h stands for the height of the measured specular surface with respect to the reference plane. Finally, ∆*d* and *d* are the distance between LCD_1_′ and LCD_2_ and the distance between the reference plane and LCD_1_′, respectively. The geometric relationship in Figure 2 gives the relations

(1) $$(\varphi_{r1}-\varphi_{r2})q/2\pi =\Delta d\cdot \tan\theta$$

(2) $$(\varphi_{m1}-\varphi_{m2})q/2\pi =\Delta d\cdot \tan\left(\theta +\Phi \right)$$

(3) (d + h)tanθ+Δl=(d−h)tan(θ+Φ)

(4) $$(\varphi_{r1}-\varphi_{m1})q/2\pi =\Delta l$$

From Equations (1)–(4), the height *h* of the measured specular surface is

(5) $$h=\frac{\Delta d\left(\varphi_{m1}-\;\varphi_{r1}\right)-d\left[(\varphi_{r1}-\;\varphi_{r2})-(\varphi_{m1}-\;\varphi_{m2})\;\right]\;}{\left(\varphi_{m1}-\;\varphi_{m2})+(\varphi_{r1}-\;\varphi_{r2}\right)}$$

Equation (5) clearly shows that height information can be directly calculated from the captured fringe patterns on the measured specular surface only if the two parameters *d* and ∆*d* and phase information on the reference plane are known beforehand. If the optimum three-fringe number selection method [30] is used to calculate the absolute phase pixel by pixel, specular objects with isolated and/or discontinuous surfaces can be measured.

According to the derived mathematical model expressed as Equation (5), the parameters of ∆*d* and *d* need to be determined accurately beforehand to obtain the 3D shape data of specular objects. Two accurate plane mirrors denoted as M_1_ and M_2_ are used to calibrate the two distances [24,25]. Mirror M_1_ has an ideal plane surface while M_2_ has a hollow ring matrix pattern with a known separation between the neighboring ring-shaped markers on the surface. Mirror M_1_ is fixed on an accurate moving stage and moved to a known position ∆*h* along the normal direction of the two LCD screens. At each mirror position, fringe pattern sets with the optimum fringe numbers are generated and displayed on LCD_2_ and LCD_1_. The displayed fringe patterns are reflected by M_1_ and captured by the CCD camera. Using the phase information from the captured fringe patterns and several known positions, parameter ∆*d* is calibrated. 

To calibrate the distance *d* between the reference plane and LCD_1_, mirror M_2_ is positioned parallel to the LCD screen and its surface is chosen as the reference plane. After calibrating the internal parameters of the CCD camera [31,32,33], the orientation of M_2_ is determined in the camera coordinate system using the hollow ring matrix pattern on the surface of M_2_ [34,35]. The same hollow ring matrix pattern is generated by software and displayed on LCD_1_ so that the CCD camera can view and capture the hollow ring matrix pattern at position LCD_1_″ (the virtual image of LCD_1_′) reflected by the surface of M_2_. The orientation of LCD_1_″ (LCD_1_′) is therefore obtained in the camera coordinate system using the hollow ring matrix pattern on LCD_1_. Distance *d* can be calculated using the obtained orientation of M_2_ and LCD_1_ in the same camera coordinate system. However, the hollow ring matrix pattern on mirror M_2_ (which is chosen as reference plane) and the LCD_1_ screen have different distances to the CCD camera, and hence they cannot be both clearly imaged and captured by the camera because of the limited DOF of the imaging lens. To solve this problem, the following section describes a new method for obtaining distance *d*.

### 2.2. Distance Calibration

**Figure 3:**
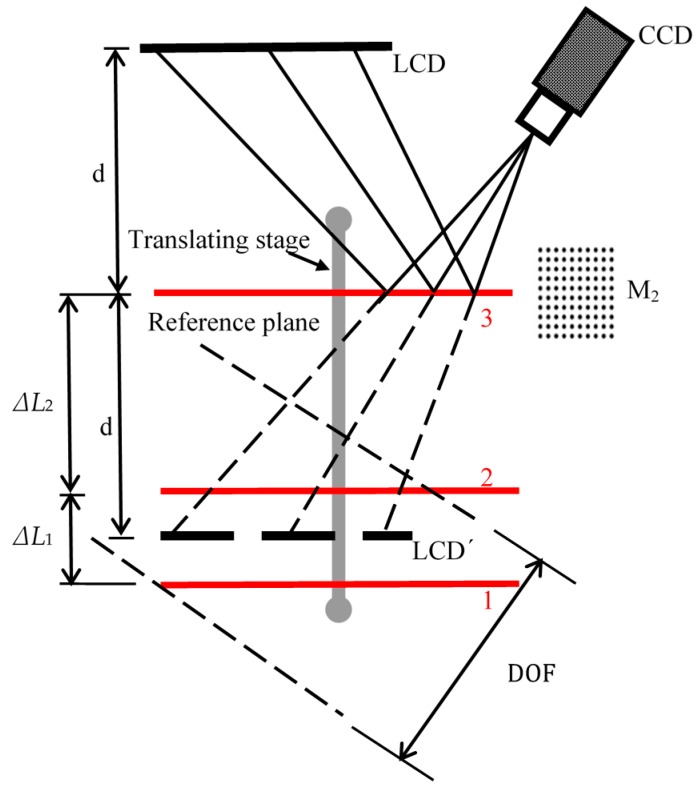
Schematic diagram for determining the stage movement direction and the distance *d* between the reference plane and the liquid crystal display (LCD) screen.

To simplify the principle of distance calibration, only the screen of LCD_1_′ is shown in the schematic diagram in Figure 3. 

#### 2.2.1. Calibration of Internal Parameters

To apply machine vision-based methods to determine distance *d* by a CCD camera, the internal parameters need to be calibrated using the following equation,

(6) $$\lambda \left[\begin{array}{c} u\\ v\\ 1 \end{array}\right]=A\left[\begin{array}{cc} R & T \end{array}\right]\left[\begin{array}{c} \begin{array}{c} X_{w}\\ Y_{w} \end{array}\\ \begin{array}{c} Z_{w}\\ 1 \end{array} \end{array}\right]$$

where *R* is a matrix representing the three rotation angles and *T* = [*t*_x_, *t*_y_, *t*_z_] is a vector representing the three linear translations. Vector [*X_w_, Y_w_, Z_w_*] is the coordinate vector of a point in the world coordinate system, while [*u*, *v*] is the coordinate vector of the corresponding point in the pixel coordinate system and λ is an arbitrary scaling factor. Matrix A consists of the CCD camera internal parameters: two focal lengths (*F_u_* and *F_v_*), two principal point coordinates (*P_u_* and *P_v_*), and four image radial and tangential distortion coefficients (*K*_1_, *K*_2_, *K*_3_, and *K*_4_). The eight internal parameters need to be calibrated beforehand and Zhang’s camera calibration method is used to obtain their values using a checkerboard at different positions [31,32,33]. 

#### 2.2.2. Stage Moving Direction in the Camera Coordinate System

The orientation of the mirror in the camera coordinate system is determined from the obtained internal parameters of the CCD camera and the known distance between the hollow ring markers. When the mirror is moved to several known positions within the DOF of the camera lens on the stage, the direction of its movement can be obtained in the camera coordinate system. At each mirror position, the hollow ring matrix is clearly imaged and captured by the CCD camera. The center position of each hollow ring on the mirror can be extracted from these captured images. Therefore, the direction of stage movement in the camera coordinate system is determined from the extracted markers and obtained internal parameters of the CCD camera. 

Figure 3 is a schematic diagram of the process to determine the stage moving direction in the camera coordinate system. Mirror M_2_ is the mirror with the hollow ring matrix pattern on the surface. When M_2_ is located at two positions (1 and 2) in the DOF of the imaging lens, the camera can clearly image and capture the hollow ring markers on the mirror surface. After extracting all the center positions of the hollow ring markers from the captured images, the orientation of the mirror in the camera coordinate system is determined by the following equations:

(7) $$A_{0}=R_{1}\times A_{1}+T_{1}\;$$

(8) $$A_{0}=R_{2}\times A_{2}+T_{2}$$

where *R*_1_ and *T*_1_ are the rotation matrix and translation vector, respectively, when the mirror is at position 1. In addition, *R*_2_ and *T*_2_ are the rotation matrix and the translation vector, respectively, when the mirror is at position 2. Vector *A*_0_ is the 3D coordinate vector of one point in the camera coordinate system, and *A*_1_ and *A*_2_ are the 3D coordinate vectors of the corresponding points in the mirror coordinate system when the mirror is at positions 1 and 2, respectively.

According to the obtained external parameters of *R*_1_, *T*_1_, and *T*_2_, the direction of the moving stage can be denoted as a vector *T*_21_ and deduced as

(9) $$T_{21}=R_{1}{}^{-1}\times \left(T_{2}-T_{1}\right)$$

(10) $$\|T_{21}\|=\Delta L_{1}$$

(11) $$\;\{\begin{array}{c} \alpha =\frac{T_{21}\left(1\right)}{\Delta L_{1}}\\ \beta =\frac{T_{21}\left(2\right)}{\Delta L_{1}}\\ \gamma =\frac{T_{21}\left(3\right)}{\Delta L_{1}} \end{array}\;$$

where ∆*L*_1_ is the moving distance of the stage, and *α*, *β*, and *γ* represent the proportion of the translation vector in the *x*, *y*, and *z* directions, respectively.

More hollow ring matrix images for different mirror positions in the DOF of the lens can be captured and used to accurately calculate the moving stage direction in the camera coordinate system.

#### 2.2.3. Distance Determination

When the mirror is moved to a new position 3 outside of the DOF of the camera lens, as illustrated in Figure 3, the CCD camera cannot clearly capture the hollow ring matrix pattern. In this case, the orientation of the mirror in the camera coordinate system is inaccurately determined by the machine vision-based method. However, if the mirror is moved to a known distance by the stage, the orientation of the mirror in the camera coordinate system can be calculated from the obtained stage’s moving direction. 

Assuming the mirror is moved a distance Δ*L*_2_ to the reference position by the moving stage, the orientation of the mirror in the camera coordinate system can be obtained using the obtained *R*_2_, *T*_2_, *α, β, γ*, and ∆*L*_2_ as

(12) $$R_{3}=R_{2}$$

(13) $$T_{3}=T_{2}+R_{2}\times T_{32}$$

(14) $$\{\begin{array}{c} T_{32}\left(1\right)=\alpha \times \Delta L_{2}\\ T_{32}\left(2\right)=\beta \times \Delta L_{2}\\ T_{32}\left(3\right)=\gamma \times \Delta L_{2} \end{array}$$

where *T*_3_ and *R*_3_ are the translation vector and rotation matrix at mirror position 3 in the camera coordinate system, respectively, and *T*_32_ is the translation vector of the mirror at its second position to the CCD camera. Because the mirror is fixed on the translation stage, Equation (12) holds when it is moved from position 2 to position 3.

When the mirror is located at position 3 (the reference plane), the virtual image LCD’ of the LCD screen reflected by the mirror is imaged in the DOF of the imaging lens. A virtual checkerboard image displayed on the LCD screen is clearly imaged and captured by the CCD camera. Because the mirror is located out of the DOF of the lens, the hollow ring matrix pattern on the mirror surface cannot be clearly imaged and captured by the CCD camera. Therefore, the phase calculation during the calibration procedure is not affected by the hollow ring matrix pattern on the mirror surface. The rotation matrix *R*_4_ and translation vector *T*_4_ of the LCD screen with respect to the CCD camera can be expressed as

(15) $$R_{4}=R_{2}$$

(16) $$T_{4}=A_{0}-R_{4}\times A_{4}$$

where, *A*_4_ represents the 3D coordinate vector of the corresponding point on the LCD screen in the screen coordinate system when the mirror is at position 3. The relative orientation between the mirror and the LCD screen has been adjusted to be parallel. The parallelism is established by repeating adjustment of the relative orientations of the three components (the two LCD screens and the mirror at reference plane) and generating pre-distorted fringe patterns for compensation. Therefore, Equation (15) holds.

Combining Equations (13) and (16), the following relationship can be deduced:

(17) $$T_{34}=R_{4}{}^{-1}\times \left(T_{3}-T_{4}\right)$$

where *T*_34_ is the translation vector between the mirror at position 3 and the virtual image of the LCD screen. Therefore, distance *d* is

(18) $$d=\|T_{34}\|$$

The obtained distance *d* can be applied to Equation (5) to calculate the 3D shape of specular surfaces.

## 3. Experiments and Results

### 3.1. Hardware System

**Figure 4:**
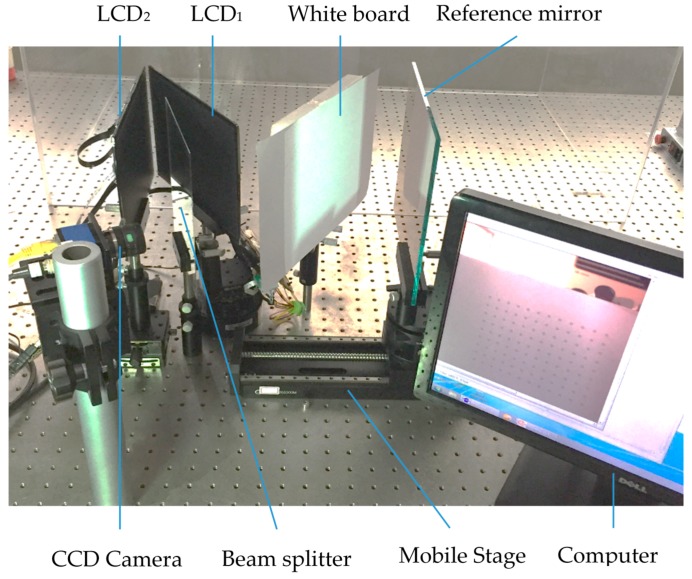
Panorama of DPMD hardware system.

To test the proposed distance calibration method, an experimental system was setup, as illustrated in Figure 4. The system consists of two 24.64 cm LCD screens with a physical resolution of 1536 × 2048 (for calibration, only was LCD_1_ used), a BS, a white board, a mirror with a hollow ring marker matrix pattern on the surface, an industrial CCD camera with a 1296 × 964 resolution, an automatic moving stage with an accuracy of 1 µm, and a PC. The BS was used to distinguish two screens; the white board is just the reflected background for capturing the hollow ring pattern on the mirror. The computer is connected to the camera and the LCD screens by a gigabit ethernet cable and HDMI (high definition multimedia interface), respectively. The computer is also used to generate and display a checkerboard pattern on the LCD_1_ screen. The CCD camera is used to capture the images of the hollow ring matrix pattern on the reference mirror and generated checkerboard displayed on the LCD_1_ screen. The LCD_1_ screen displays the checkerboard pattern and generated fringe patterns. The moving stage moves the mirror to several known positions for determining the orientation of the mirror in the camera coordinate system. The mirror is used to calibrate the stage moving direction in the camera coordinate system and reflect the checkerboard pattern displayed on the LCD_1_ screen. LCD_2_ is used to obtain data during the object measurement; it is not necessary during distance calibration. 

It is assumed that the mirror is parallel to the LCD screens in the experimental system. This assumption can be satisfied using a software-based method as follows. First, the intrinsic parameters of the CCD camera are calibrated by imaging a checkerboard from several viewpoints. Then, a picture of the checkerboard is generated by software, displayed on the LCD screen, and captured by the CCD camera. The size of the checkerboard can be obtained because the unit pixel size of the LCD is known. Finally, the external parameters (*R* and *T* matrices) of the camera coordinate system and angle between the camera and target surface of the camera in the *x*, *y*, and *z* directions can also be determined. Therefore, they provide a basis for the parallel adjustment. 

### 3.2. Experimental Process and Results

**Figure 5:**
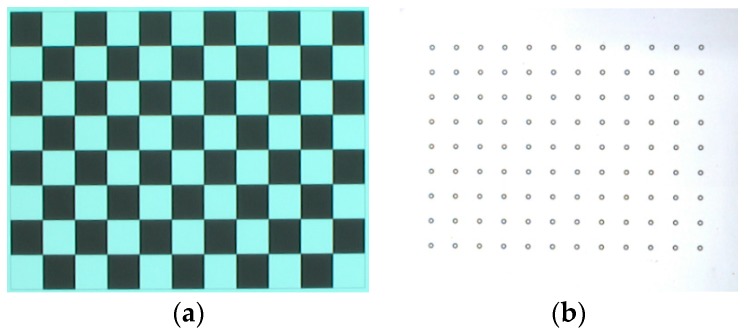
Photo of the calibration targets. (**a**) Checkerboard; (**b**) mirror with a hollow ring matrix pattern on its surface.

**Figure 6:**
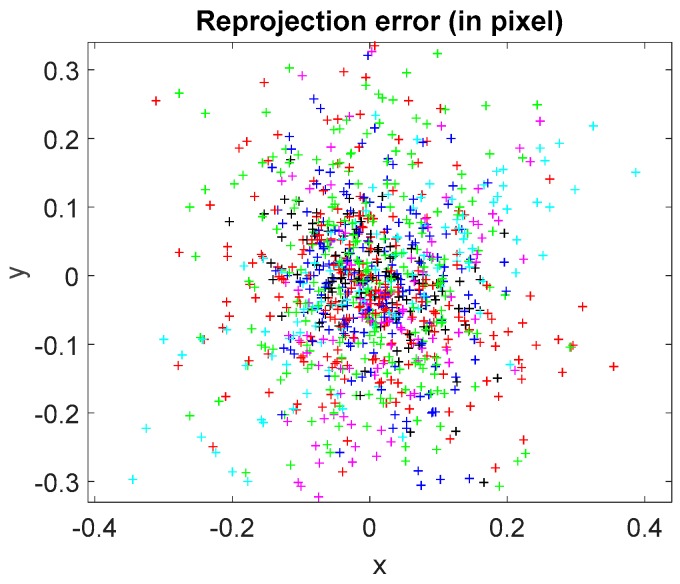
Reprojection error of the calibrated camera.

A checkerboard with 9 × 12 checkers and a mirror with a matrix pattern of hollow ring markers on the surface were designed and manufactured by Ti-Times [36] and Giai Photonics Co., Ltd. [37], respectively, as illustrated in Figure 5. The checker size along the row and column directions has the same value of 6 mm. The distance between the adjacent hollow ring markers along horizontal and vertical directions is 6 mm. To accurately determine the internal parameters of the CCD camera, the checkerboard was positioned at 28 random positions and orientations with a large angle between the imaging axis and the board’s normal. At each position, the CCD camera captured the board image. Using the known checker size in the eighteen captured images, the internal parameters of the CCD camera were obtained by the Camera Calibration Toolbox for MATLAB [32]. The radial and tangential distortions of the following captured images were corrected by the obtained internal parameters. The average reprojection errors in the *x* and *y* directions are 0.150 pixels and 0.230 pixels, respectively, as illustrated in Figure 6.

**Figure 7:**
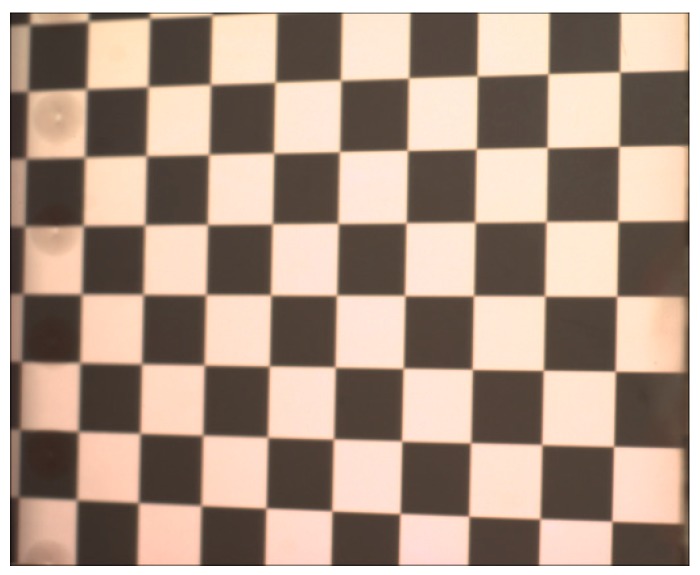
Captured virtual checkerboard image reflected by the mirror.

After determining the internal parameters of the CCD camera, distance *d* was calibrated using the proposed method. First, the mirror with the hollow ring matrix pattern was fixed on the stage and adjusted so that the surface was perpendicular to the moving direction. The initial position of the mirror was in the DOF of the camera lens. The CCD camera clearly captured the hollow ring matrix pattern. Second, the mirror was moved 12.001 mm by the moving stage. This position was still within the DOF of the camera lens and the hollow ring matrix pattern was clearly captured by the camera. The moving direction of the stage in the camera coordinate system was calculated using the method proposed in Section 2.2.2. Third, the stage moved the mirror 128.000 mm to a new position, which was out of the DOF of the camera lens. In this situation, the hollow ring matrix pattern is out of focus and cannot be clearly captured by the camera. The orientation of the mirror was calculated by the known moved distance and calculated moving direction. Fourth, a checkerboard image was generated and displayed on the LCD_1_ screen. The virtual checkerboard reflected by the mirror was located in the DOF and captured by the CCD camera. Because the mirror was out of the DOF of the imaging lens, the captured virtual checkerboard image reflected by the mirror clearly shows the checker without the hollow ring matrix pattern, as illustrated in Figure 7. The orientation of the LCD_1_ screen was calculated using the checker size and obtained internal parameters of the CCD camera. Finally, the distance between the mirror at the reference position and the LCD_1_ screen was obtained as 136.742 mm in the established DPMD system because the two components are parallel in the same camera coordinate system. 

### 3.3. Performance Analysis

To evaluate the proposed method, two techniques were applied to verify the accuracy of the calibrated distance. One directly relates to a known distance moved by the moving stage; the other performs indirect verification by measuring an artificial step in the established DPMD system. Because the distance the mirror is moved by the stage is accurately known beforehand, it can be directly used to verify the calibrated distance. The accuracy can be acquired by comparing the distance moved with the distance calibrated by the proposed method, as shown in Table 1. The maximum value of the absolute error is less than 0.030 mm.

**Figure 8:**
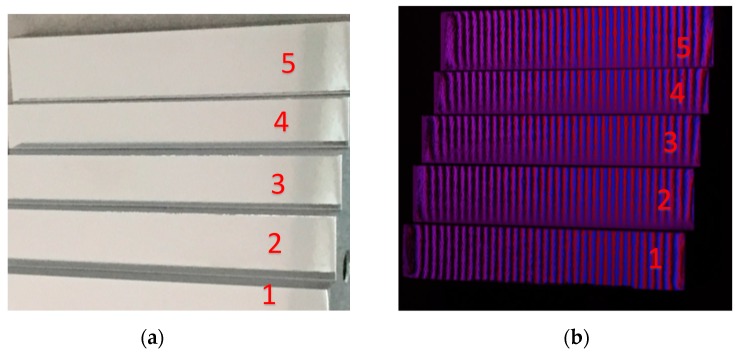

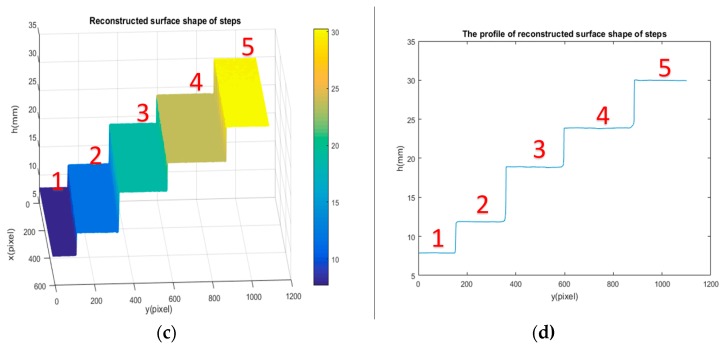
Illustration of the designed step-shaped mirror with step numbers and measured depths. (**a**) The designed steps; (**b**) image of the fringe pattern reflected by the stepped surface; (**c**) shaded display of the measured depth; (**d**) the profile of the measured mirror step. The *X*- and *Y*-axes represent the pixel positions.

An artificial stepped mirror was designed and manufactured to indirectly verify the accuracy of the calibrated distance, as illustrated in Figure 8. This step was measured by reflecting the fringe patterns and captured by the CCD camera, as shown in Figure 8b. Figure 8c, d illustrates shading display and profile of the measured depth data from the calibrated system. The actual and measured distance between the neighboring steps as well as absolute error (absolute difference between the measured average distance and actual distance) are listed in Table 2. The maximum absolute error is 0.031 mm. 

**Figure 9:**
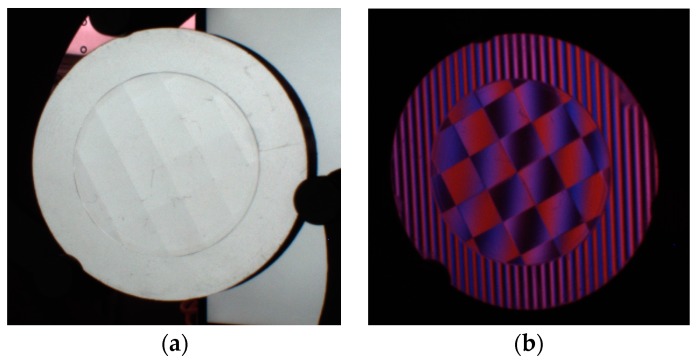
The reflected diamond distribution surface object. (**a**) The photo of the object; (**b**) image of the fringe pattern reflected by the diamond distribution surface object.

A reflected diamond distribution surface (DDS) object was also measured by the developed 3D measurement system, as illustrated in Figure 9.

**Figure 10:**
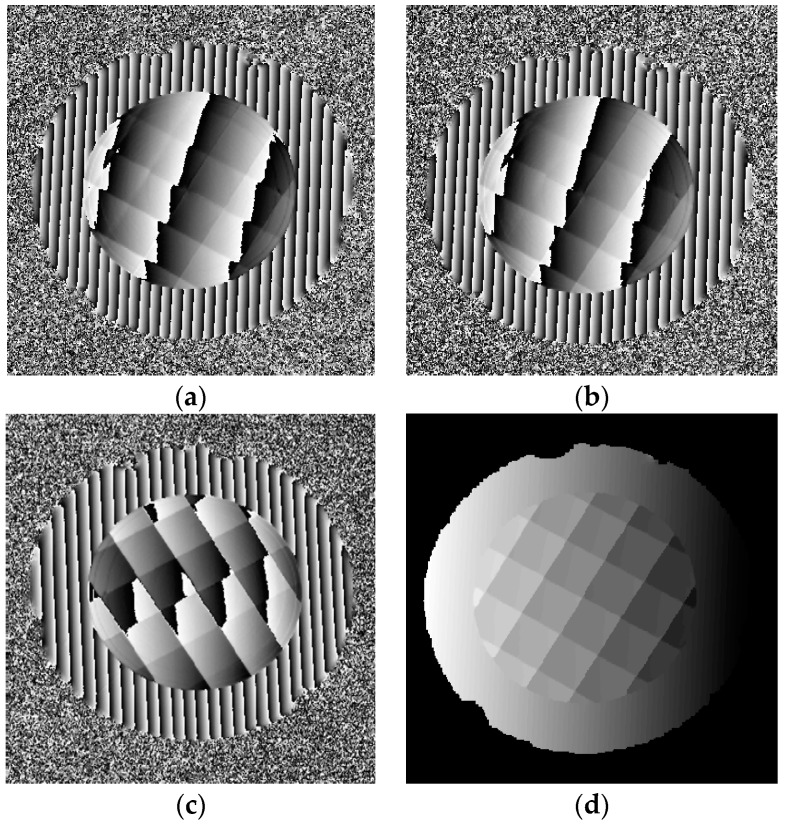
Phase maps of the reflected diamond distribution surface object. (**a**–**c**) are three wrapped phase maps having fringe numbers of 100, 99 and 90, (**d**) is the absolute phase map.

Twelve sinusoidal fringe patterns having optimum fringe numbers of 100, 99 and 90 were generated by software and sequentially displayed on the two LCD screens. Fringe patterns reflected by the specular surface of the DDS object were deformed and captured by the triggered CCD camera from another viewpoint, as illustrated in Figure 9b. Using four-step phase-shifting algorithm, three wrapped phase maps were calculated for the measured specular object, as shown in Figure 10a–c. The absolute phase of each pixel was determined using the optimum three-fringe number selection method, as shown in Figure 10d. 

**Figure 11:**
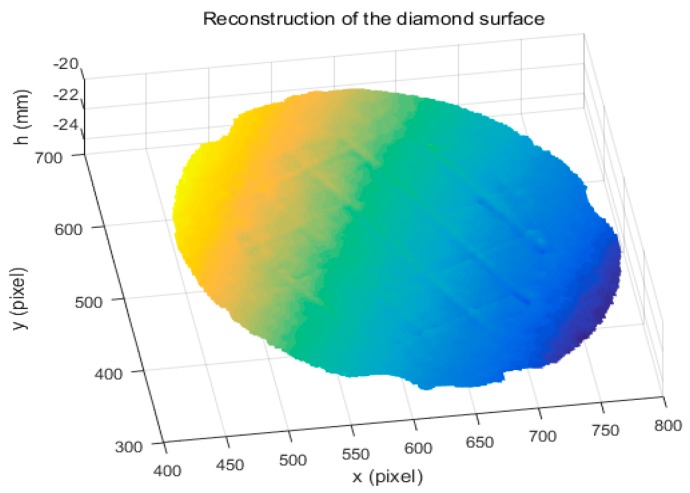
Reconstruction of the reflected diamond distribution surface object.

Using the calibrated parameters ∆*d* and *d*, the reconstruction of DDS data was obtained, as shown in Figure 11. The results show that the proposed method can directly measure a specular object having isolated and/or discontinuous surfaces. 

## 4. Conclusions

This paper presented a novel method for calibrating the distance *d* between a mirror at the reference plane and the LCD screen for the DPMD system. The mirror and the LCD screen cannot be clearly captured given the limited depth of field (DOF) of an imaging lens because of their large depth distance along optical axis. To solve the problem caused by the limited DOF of the imaging lens, a novel calibration method by using an automatic moving stage has been presented to determine the orientation of the mirror in the camera coordinate system. Mathematical derivation procedure was deliberated to obtain the distance *d*. As a result, the distance between the mirror and the LCD screen was accurately calibrated for the DPMD system. The experimental results obtained using direct and indirect measurements show that the proposed method can accurately obtain this distance. The maximum value of the absolute error is less than 0.031 mm by using the proposed method. In fact, the proposed method solves two problems. One problem is the limited DOF of the imaging lens, which means that the hollow ring matrix pattern on the mirror surface cannot always be clearly imaged, causing the calibrated distance value to be inaccurate. In the proposed method, *d* can accurately be determined. The other is the effect of the hollow ring matrix pattern on the identification of the checker corners and calibration of the DPMD system. Because the mirror is out of the DOF of the camera lens, the hollow ring matrix pattern is not visible in the reflected virtual checkerboard image captured by the CCD camera. Therefore, the mirror with the hollow ring matrix pattern can also be used as a common mirror without any markers on the surface. 

The proposed calibration method is implemented in the lab because of the usage of a translating stage. Therefore, a simple and flexible calibration method needs to be developed in the near future. The other challenging problems in PMD or DPMD are measuring dynamic specular surface, some components having specular and non-specular parts on the same object surface, and so on. A possible solution is to combine interferometry into deflectometry to significantly improve the precision with a large dynamic range. 

## Figures and Tables

**Table 1:** Direct test of the calibrated distance using the moving stage at the positions of 8 mm, 8.5 mm, 10 mm 11.5 mm, and 12 mm (Unit: mm).

Position	8	8.5	10	11.5	12
Moved distance	278.783	279.249	280.485	281.854	282.324
Calibrated distance	278.762	279.219	280.512	281.879	282.352
Absolute error	0.021	0.030	0.027	0.025	0.028

**Table 2:** Experimental results on the mirror step artifact (Unit: mm).

Depth	Step Distance	Measured Distance	Absolute Error	RMS
step 1 to 2	3.987	4.018	0.031	0.030
step 2 to 3	7.025	7.046	0.021	0.013
step 3 to 4	5.006	4.986	0.020	0.023
step 4 to 5	6.099	6.075	0.024	0.020
